# EDS1-Dependent Cell Death and the Antioxidant System in *Arabidopsis* Leaves is Deregulated by the Mammalian Bax

**DOI:** 10.3390/cells9112454

**Published:** 2020-11-10

**Authors:** Maciej Jerzy Bernacki, Weronika Czarnocka, Magdalena Zaborowska, Elżbieta Różańska, Mateusz Labudda, Anna Rusaczonek, Damian Witoń, Stanisław Karpiński

**Affiliations:** 1 Institute of Technology and Life Sciences, Falenty, Al. Hrabska 3, 05-090 Raszyn, Poland; mjbernacky@gmail.com; 2 Department of Plant Genetics, Breeding and Biotechnology, Institute of Biology, Warsaw University of Life Sciences, Nowoursynowska Street 159, 02-776 Warsaw, Poland; weronika_czarnocka@sggw.edu.pl (W.C.); magdazaborowskaa@gmail.com (M.Z.); anna_rusaczonek@sggw.edu.pl (A.R.); damiankamilwiton@gmail.com (D.W.); 3 Department of Botany, Institute of Biology, Warsaw University of Life Sciences, Nowoursynowska Street 159, 02-776 Warsaw, Poland; elzbieta_rozanska@sggw.edu.pl; 4 Department of Biochemistry and Microbiology, Institute of Biology, Warsaw University of Life Sciences, Nowoursynowska Street 159, 02-776 Warsaw, Poland; mateusz_labudda@sggw.edu.pl

**Keywords:** cell death, Bax, EDS1, ROS, hypersensitive response

## Abstract

Cell death is the ultimate end of a cell cycle that occurs in all living organisms during development or responses to biotic and abiotic stresses. In the course of evolution, plants and animals evolve various molecular mechanisms to regulate cell death; however, some of them are conserved among both these kingdoms. It was found that mammalian proapoptotic BCL-2 associated X (Bax) protein, when expressed in plants, induces cell death, similar to hypersensitive response (HR). It was also shown that changes in the expression level of genes encoding proteins involved in stress response or oxidative status regulation mitigate Bax-induced plant cell death. In our study, we focused on the evolutional compatibility of animal and plant cell death molecular mechanisms. Therefore, we studied the deregulation of reactive oxygen species burst and HR-like propagation in *Arabidopsis thaliana* expressing mammalian Bax. We were able to diminish Bax-induced oxidative stress and HR progression through the genetic cross with plants mutated in *ENHANCED DISEASE SUSCEPTIBILITY 1* (*EDS1*), which is a plant-positive HR regulator. Plants expressing the mouse Bax gene in *eds1-1* null mutant background demonstrated less pronounced cell death and exhibited higher antioxidant system efficiency compared to Bax-expressing plants. Moreover, *eds1*/Bax plants did not show HR marker genes induction, as in the case of the Bax-expressing line. The present study indicates some common molecular features between animal and plant cell death regulation and can be useful to better understand the evolution of cell death mechanisms in plants and animals.

## 1. Introduction

Cell death is a process occurring in all multicellular organisms. It is crucial in the development senescence and growth of both animals and plants [1,2]. Moreover, it is important in response to various stress factors and is engaged in different types of diseases and immune defence [3] and in acclimatory responses [4]. In the course of evolution, plants and animals have evolved various genetic and molecular mechanisms to regulate cell death [5,6]. However, some of the molecular pathways are conserved between these two kingdoms [6,7,8], and some homologs of animal apoptotic genes are present also in plant genomes [9].

The best described mammalian apoptotic proteins belong to the Bcl-2 family [10,11]. The Bcl-2family contains both pro- and antiapoptotic regulators that possess Bcl-2 Homology (BH) domains [10,11]. One of the proapoptotic proteins from this family is Bcl-2-associated X (Bax) protein [12]. Activation of the Bax protein is a result of a highly regulated multistep process and leads to its translocation from the cytoplasm to the mitochondrial outer membrane (MOM) [13]. The location of the Bax protein in MOM allows the outflow of soluble proteins, including cytochrome C, from the mitochondria to cytosol, where they activate caspases, which further leads to apoptosis initialization [11,14,15]. The Bcl-2 family proteins are absent in yeasts and plants [9]. However, when the gene encoding mammalian Bax is artificially expressed in plants or yeast, it results in cell death [7,8,16,17]. Similarly to animal cells, the Bax protein, introduced to plant cells, is located in the mitochondrial membrane [8,17], and its transmembrane domain is necessary for lethal effect [8]. It was found that Bax-induced plant cell death in *Nicotiana benthamiana* is similar to the hypersensitive response (HR). The Bax-promoted cell death phenotype in *Nicotiana benthamiana* strongly resembled the HR phenotype induced in wildtype plants by tobacco mosaic virus. It was also shown that, during Bax-induced plant cell death, there is an accumulation of PATHOGENESIS-RELATED PROTEIN 1 (PR1) [8]. Although the proteins belonging to the Bcl-2 family are absent in plants, a homologue of Bax Inhibitor-1 (BI-1) was found in *Arabidopsis thaliana* [18]. BI-1 is an attenuator of cell death progression, induced by both biotic and abiotic stress. *Arabidopsis thaliana atbi1-1* and *atbi1-2* mutants are more susceptible to pathogens and heat stress than the wildtype [19]. Moreover, it was found that the *BI-1* expression level was upregulated in response to the abovementioned factors in wildtype plants [19]. Overexpression of *BI-1* resulted in reversion of the runway cell death phenotype in the *lesion simulating disease 1* (*lsd1-1*) mutant [20], probably by its role in membrane stabilization and in deregulation of Ca^2+^ flux in the endoplasmic reticulum and chloroplast retrograde signalling during stress [21,22]. In Arabidopsis plants expressing Bax gene under a dexamethasone (DEX)-inducible promoter, the addition of DEX to the growth medium was sufficient to evoke cell death [7,23]. Interestingly, plants expressing both Bax and BI-1 survived and could continue to grow in a medium containing DEX [7], but they demonstrated no differences in PR1 mRNA accumulation level in comparison to Bax-expressing plants [7]. Moreover, Bax-induced plant cell death turned out to be reactive oxygen species (ROS)-dependent [17]. It was shown that the enhancement of ROS metabolism/decomposition by overexpressing *NUCLEOSIDE DIPHOSPHATE KINASE 2* (*NDPK2*) or by strong antioxidant treatment resulted in partial cell death inhibition in Bax-expressing plants [17].

In the context of Bax-induced deregulation of plant cell death, the ENHANCED DISEASE SUSCEPTIBILITY 1 (EDS1) protein seems to be interesting. EDS1 together with its interacting partner, PHYTOALEXIN DEFICIENT 4 (PAD4), was described as a crucial regulator of salicylic acid (SA)-dependent defence response to biotic stresses [24,25] and as an HR regulator functioning upstream of SA-dependent *PR1* mRNA accumulation [26]. Later on, it was demonstrated that SA- and EDS1-dependent HRs in plants are also regulated by chloroplast retrograde signalling depending on non-photochemical quenching (NPQ), the redox status of the plastoquinone pool and photooxidative ROS burst, and ethylene accumulation [22]. Moreover, it was shown that EDS1 is important in response to various abiotic stress [20,27,28,29]. Therefore, EDS1 is now considered a nonspecific, stress-related protein [22]. Both EDS1 and PAD4 exhibit homology to eukaryotic acyl lipases, and the EDS1-PAD4 complex is required for SA, ethylene, and ROS accumulation [26,30,31]. EDS1 forms dimers also with SENESCENCE-ASSOCIATED GENE 101 (SAG101) and LESION SIMULATING DISEASE 1 (LSD1) [31,32,33,34,35]. It was shown that EDS1 is mostly present in the cytoplasm while the EDS1-LSD1 and EDS1-PAD4 dimers are present both in the cytoplasm and nucleus [31,32,35]. The nucleus–cytoplasm ratio of EDS1 seems to be very important in plant immunity [35]. It was postulated that EDS1 together with LSD1 and PAD4 constitute a molecular hub which conditionally regulates signal transduction pathways, hormonal homeostasis, cell death, and acclamatory and defence responses in Arabidopsis [21,22,36].

In the current study, we wanted to test if the mammalian Bax can deregulate the EDS1-dependent cell death mechanism [21,22,36]. Therefore, we crossed the *eds1* mutant with the transgenic plants harbouring the Bax construct under a DEX-induced promoter. We showed that Bax-induced HR-like response (cell death) and growth arrest can be almost completely attenuated in the *eds1* mutant background. Our result also suggests that mammalian Bax destabilizes organelle membranes in plants like it does in animals during cell death induction. Moreover, ROS and hormonal burst, normally induced in wildtype plants (with functional EDS1) are required for HR-like cell death induction in Arabidopsis expressing mammalian Bax. Our results suggest that various molecular elements of cell death mechanisms in plants and animals are compatible and, therefore, could evolve before separation of these two kingdoms.

## 2. Materials and Methods

### 2.1. Plant Material and Growth Conditions

*Arabidopsis thaliana eds1* mutant plants harbouring Bax under the control of the dexamethasone (DEX)-induced promoter [7] were used in this study together with Columbia-0 (Col-0) plants. Seeds of the Bax line were a kind gift of Prof. Maki Kawai-Yamada. The *eds1/*Bax line was obtained in our laboratory by crossing. Homozygosity of the F3 generation was checked on Murashige–Skoog medium containing hygromycin (20 μg mL^−1^) and using real-time qPCR (Figure S1A,B). Plants where grown in the growing chamber under the following conditions: 8/16 h photoperiod, photosynthetic photon flux density (PPFD) of 130 μmol m^−2^ s^−2^, air humidity 60%, and temperature 20/18 °C day/night.

### 2.2. Induction of the Bax Gene

The Arabidopsis Bax-expressing line and *eds1/*Bax plants possess the open reading frame (ORF) of the mouse Bax gene under the dexamethasone (DEX)-inducible promoter in the vector pTA7002. During the fourth week of growth, plants were sprayed with 1 mM DEX solution or with the control solution (2 mL 96% ethanol + 48 mL tap water) three times in one-day intervals. All measurements were made two days after the last spray application.

### 2.3. Biometric Measurements

The dry mass was weighed using a laboratory scale (Sartorius Lab Instruments GmbH & Co. KG, Göttingen, Germany). The rosette area was measured using the Fluor Cam 800 MF PSI device (PSI Photon Systems Instruments, Brno, Czech Republic).

### 2.4. Relative Electrolyte Leakage

Each Arabidopsis rosette was decapitated and placed in a 50-mL falcon tube filled with 30 mL of Milli-Q water (Merck Millipore, Darmstadt, Germany). Immediately after rosette submergence, the electrolyte leakage was measured (T0) with a conductance meter (Xylem Analytics Germany Sales GmbH & Co. KG WTW, Weilheim, Germany). After 1 h, the electrolyte leakage was measured again (T1). Then, in order to obtain complete ion leakage, the falcon tubes were frozen in −80 °C for 24 h. After thawing, electrolyte leakage was measured again (T2). The relative electrolyte leakage was calculated as a ratio between the T1–T0 value and T2 value. The relative electrolyte leakage was expressed as a percent of total electrolyte leakage.

### 2.5. Trypan Blue Staining

Prior to measurements, the leaf discs were cut from the seventh leaf from at least five different plants per genotype. Discs were submerged in a staining solution (0.016% trypan blue, 8% phenol, 8% glycerol, 8% lactic acid, and 65% ethanol) and incubated in 100 °C for 3 min. The samples were then incubated overnight in room temperature. Next, the samples were decolorized in 6 M chloral hydrate solution. Dead cells (coloured with blue) were observed, and the pictures were taken using Leica M165-FC fluorescent stereo microscope (Leica Microsystems, Wetzlar, Germany).

### 2.6. RNA Isolation, cDNA Synthesis, and Quantitative Real-Time PCR

Rosettes were frozen in liquid nitrogen in three biological replicates, each containing 8–12 plants. About 100 mg of tissue was weighed and subjected to RNA isolation using Spectrum™ Plant Total RNA Kit (Sigma Aldrich, St. Louis, MO, USA). The obtained RNA was purified from residual DNA with the Ambion Turbo DNAse kit (Thermo Fisher Scientific, Waltham, MA, USA) according to the manufacturers’ protocol. The concentration of RNA was measured using a UV-Vis spectrophotometer NanoDrop™ (Thermo Fisher Scientific, Waltham, MA, USA), and the RNA was diluted in order to obtain the same concentration in all tubes. cDNA was synthesized on 2 µg of total RNA using a High-Capacity cDNA Reverse Transcription Kit (Thermo Fisher Scientific, Waltham, MA, USA). qPCRs were performed in three technical repetitions for each of the three biological replicates using the Power SYBR Green PCR Master Mix and the ABI 7500 Fast Real-Time PCR System (Thermo Fisher Scientific, Waltham, MA, USA). Two reference genes were used, according to the RefGenes tool incorporated in Genevestigator [37], *5-FORMYLTETRAHYDROFOLATE CYCLOLIGASE* (*5-FCL*, AT5G13050), and *PROTEIN PHOSPHATASE 2A SUBUNIT A2* (*PP2AA2*, AT3G25800). All primers used in this work are listed in Table S1. Specificity of the amplified PCR products was verified by melting curve analysis. Reaction efficiency was calculated using LinRegPCR [38]. The statistical significance of differences in all tested gene expressions among tested lines was calculated using REST2009 [39].

### 2.7. Anatomic and Ultrastructural Analysis

From each tested genotype, the seventh leaf was dissected in 3 biological replicates, fixed in 2% (*v*/*v*) glutaraldehyde (Sigma Aldrich, St. Louis, MO, USA) and 2% (*w*/*v*) paraformaldehyde (Sigma Aldrich, St. Louis, MO, USA) in 50 mM sodium cacodylate buffer (pH 7.2) (Sigma Aldrich, St. Louis, MO, USA) for 2 h. Afterwards, leaves were washed three times for 10 min with 50 mM cacodylic buffer and embedded in epoxy resin, as described previously [40]. Leaf segments were sectioned on a Leica RM2165 microtome (Leica Microsystems, Wetzlar, Germany) into 3-µm thick sections that were collected on glass slides, stained with 1% (*w*/*v*) aqueous solution of crystal violet dye (Sigma-Aldrich, St. Louis, MO, USA) and examined in an Olympus AX70 “Provis” light microscope (Olympus, Tokyo, Japan) equipped with an Olympus DP50 digital camera (Olympus, Tokyo, Japan). At select places, ultrathin sections (90 nm thick) were taken for transmission electron microscopy with a Leica UCT ultramicrotome (Leica Microsystems, Wetzlar, Germany). Ultrathin sections were stained with saturated solution of uranyl acetate (Sigma Aldrich, St. Louis, MO, USA) followed by incubation in lead citrate (Sigma Aldrich, St. Louis, MO, USA) and examined in an FEI 268D “Morgagni” transmission electron microscope (FEI Company, Hillsboro, OR, USA) equipped with an Olympus-SIS “Morada” digital camera (Olympus, Tokyo, Japan). Collected digital microscopic images were processed for similar contrast and brightness with Adobe Photoshop software. Samples were collected in three independent experiments.

### 2.8. Protein Extraction

Protein extracts were prepared as previously described [41]. Briefly, rosettes were grinded in a mortar with an ice-cold extraction buffer containing 50 mM potassium phosphate buffer (pH 7.0), 2 mM 2-mercaptoethanol, 0.1 mM ethylenediaminetetraacetic acid (EDTA), 0.5% (*v*/*v*) Triton X-100, 2% (*w*/*v*) polyvinylpyrrolidone, and 1 mM phenylmethylsulfonyl fluoride (PMSF). Homogenates were incubated on ice for 20 min and centrifuged (4 °C, 20 min, and 16,000 *g*). Obtained supernatants were used to determine total soluble protein content and processed further to measure antioxidant enzyme activities.

### 2.9. Total Soluble Protein Content

Measurements were conducted using a Coomassie Brilliant Blue G-250 stain according to [42], with bovine serum albumin as a protein standard. The total soluble protein content was expressed in mg per gram of fresh weight (FW).

### 2.10. Measurements of Antioxidant Enzyme Activity

Superoxide dismutase (SOD) activity was measured according to the method described before [43]. An assay buffer was prepared by mixing equal volumes of 67 mM K/Na phosphate buffer (pH 7.8) and 25 mM EDTA. The pH value of this solution was adjusted to 10 by tetramethylethylenediamine (TEMED). Then, 1 mL of the assay buffer was added to 0.1 mL of the supernatant (previously diluted with Milli-Q water, 1:100). The SOD enzymatic assay was initiated by the addition of 0.1 mL of 2.5 μM quercetin in dimethyl sulfoxide (DMSO). The absorbance of samples at 406 nm was recorded immediately and again after 20 min. SOD activity was expressed in arbitrary units (the amount of SOD that inhibits superoxide-driven oxidation of quercetin by 50%) per gram of FW.

### 2.11. H_2_O_2_ Measurement and DAB Staining

The H_2_O_2_ level and DAB (3,3′-diaminobenzidine) staining was measured/performed as described before [20,44].

### 2.12. Chlorophyll a Fluorescence Measurements

The pulse amplitude-modulated method of chlorophyll *a* (chl *a*) fluorescence measurement implemented into a FluorCam 800 MF PSI device (Photon Systems Instruments, Brno, Czech Republic) was used as an indicator of the activity of photosystem II (PSII). Eighteen to twenty-two *Arabidopsis thaliana* plants were used for measurement for each genotype for each treatment (+mock/+DEX). Plants used for PSII activity measurement were grown under the conditions described above and were adapted in the dark 30 min before measurement. The following parameters were measured: quantum yield of PSII photochemistry in the dark-adapted state (Fv/Fm), maximum quantum efficiency of PSII photochemistry (Fv’/Fm’), effective quantum yield of PSII photochemistry (ϕPSII), and non-photochemical quenching (NPQ).

## 3. Results

### 3.1. Artificially Bax-Induced HR-Like Cell Death Propagation and Growth Inhibition Depend on EDS1

**Figure 1:**
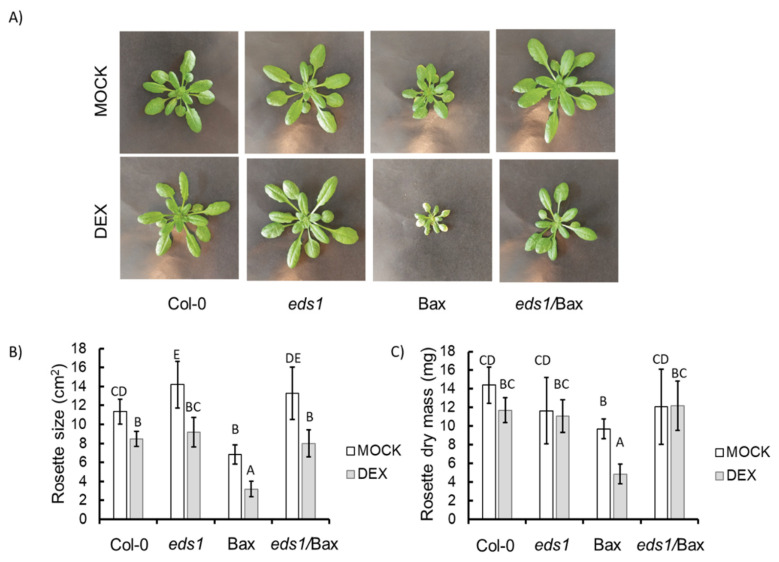
Plant phenotype (**A**), rosette size (**B**), and biomass (**C**) in the wildtype, *ENHANCED DISEASE SUSCEPTIBILITY 1* (*eds1*) mutants, and Bax and *eds1*/Bax lines (+mock/+DEX (dexamethasone)): within a subgraph, values sharing common labels (letters) are not significantly different from each other (*p* > 0.001) (*n* = 8–12).

**Figure 2:**
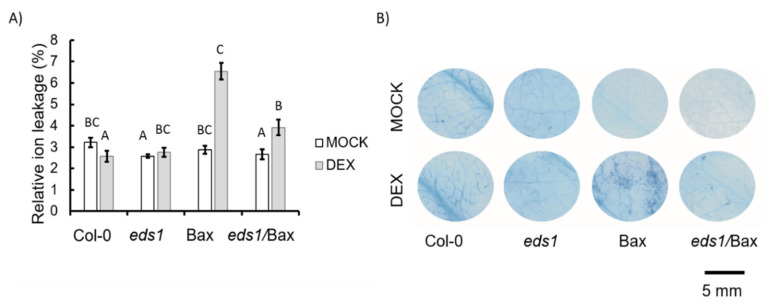
Cell death in the wild type, *eds1* mutants, and Bax and *eds1*/Bax lines (+mock/+DEX) shown as (**A**) ion leakage and (**B**) trypan blue staining of dead cells: within a subgraph, values sharing common labels (letters) are not significantly different from each other (*p* > 0.001) (*n* = 10–18).

It is known that Bax can induce the cell death mechanism in plants, and our goal was to check if EDS1-dependent signalling is involved in this process. Therefore, the *eds1* mutant was crossed with plant harbouring the Bax gene under the DEX-inducible promoter (Figure S1 and [7]). The obtained *eds1*/Bax line was widely tested in the context of plant morphology and cell death. Even among the plants treated with the mock solution, we found some differences in growth. The Bax plants (+mock) were significantly smaller (Figure 1A,B) and had reduced biomass (Figure 1C) when compared to the mock-treated wildtype plants and other mutants used in this study. Among plants treated witch DEX solution, we found even more pronounced differences. The Bax (+DEX) plants were significantly smaller, and their biomass was lower than the wildtype and *eds1* mutant (+DEX) (Figure 1A–C), while *eds1*/Bax (+DEX) plants did not differ from Col-0 (Figure 1A–C). This effect was reproducible and observed in all individual plants (Figure S2). Moreover, we found significant differences in cell death among the tested genotypes. Within mock-treated plants, Bax line did not demonstrate significant differences in ion leakage (Figure 2A). However, the *eds1* mutant and *eds1*/Bax line were proven to have lower ion leakage compared to the wildtype (Figure 2A). Among plants treated with DEX, we found notable changes in cell death level. The DEX-treated wildtype and *eds1* mutant did not exhibit higher ion leakage, while in Bax plants + DEX, we found two times higher ion leakage when compared to the wildtype + DEX. Ion leakage in *eds1*/Bax + DEX plants was slightly increased in comparison to the wildtype + DEX but was clearly lower than in *Bax* + DEX (Figure 2A). Using trypan blue staining, we found very few dead cells among plants treated with the mock solution (Figure 2B). Similarly, in the DEX-treated wildtype and *eds1* mutant, the number of dead cells was small (Figure 2B). However, in Bax + DEX plants, we found a relatively high number of dead cells (Figure 2B), while cell death was evidently less pronounced in *eds1*/*Bax* + DEX plants when compared to Bax line (Figure 2B). Here, we clearly show that EDS1-dependent molecular cell death mechanisms can be deregulated by animal Bax since Bax-dependent growth inhibition and Bax-induced HR-like cell death propagation were reverted in the *eds1* null mutant background.

### 3.2. EDS1-Dependent Regulation of Antioxidant System and ROS Metabolism is Required for Bax-Induced HR-Like Cell Death

**Figure 3:**
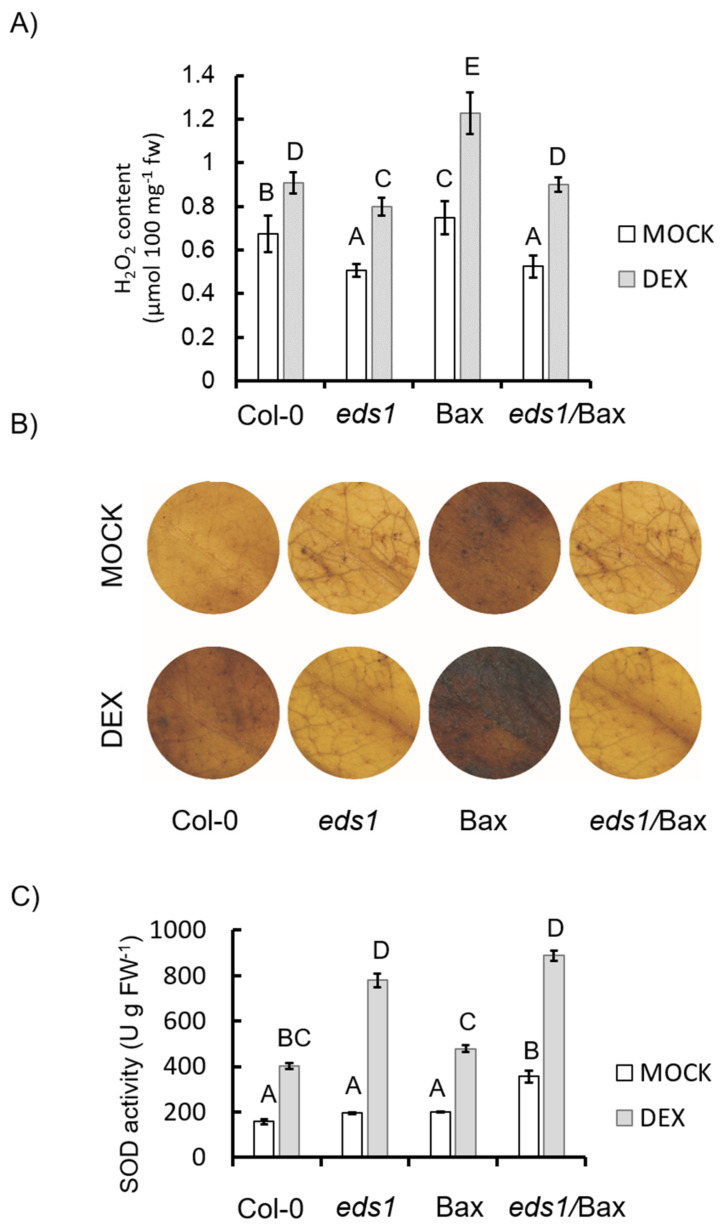
Content of H_2_O_2_ and superoxide dismutase (SOD) activity in the wildtype, *eds1* mutant, and Bax and *eds1*/Bax lines (+mock/+DEX): (**A**) H_2_O_2_ content in leaves, (**B**) H_2_O_2_ visualization using DAB (3,3′-diaminobenzidine) staining, and (**C**) activity of SOD. Within a subgraph, values sharing common labels (letters) are not significantly different from each other (*p* > 0.001) (*n* =10–12).

**Figure 4:**
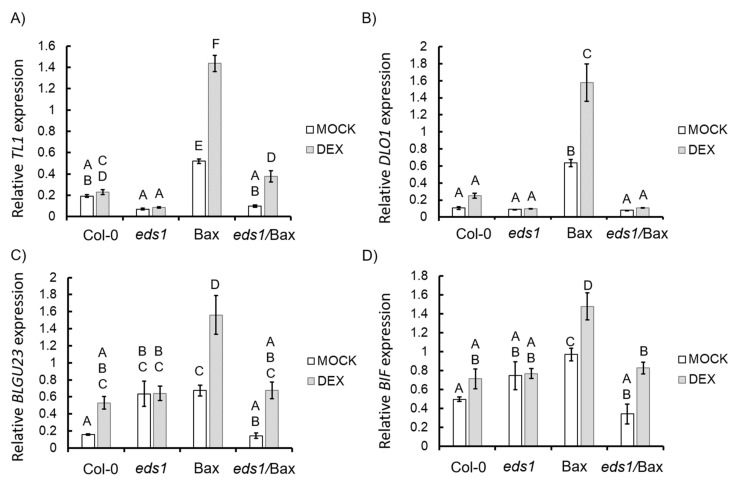
Relative expression level of marker genes of different reactive oxygen species (ROS) forms in the wild type, *eds1* mutant, and Bax and *eds1*/Bax lines (+mock/+DEX): marker gene for (**A**) many ROS forms (*TL1*), (**B**) hydrogen peroxide (H_2_O_2_) (*DLO1*), and superoxide (O_2_^−^) (**C**) *BLGU23* and (**D**) *BIF*). Within a subgraph, conditions sharing common labels (letters) are not significantly different from each other (*p* > 0.001) (*n* = 8–10).

It is well known that ROS are crucial molecules in cell death signalling [45,46], and it was shown many times that EDS1 is an important ROS homeostasis regulator in plants [20,22,27,47]. Moreover, it was found that the expression of mammalian BAX in plants leads to higher ROS production [17]. Therefore, we decided to perform extensive analyses of ROS homeostasis and the antioxidant system. In the group of plants treated with the mock solution, we found 25% lower H_2_O_2_ content in *eds1* and *eds1*/Bax plants and 15% higher H_2_O_2_ content in Bax plants in comparison to the wild type (Figure 3A). In the group of plants treated with the DEX solution, we found even more pronounced differences. Similar to mock-treated plants, DEX-treated *eds1* proved to have lower H_2_O_2_ foliar concentration compared to the wildtype. The Bax + DEX plants exhibited the highest H_2_O_2_ content, whereas the level of H_2_O_2_ in *eds1*/Bax + DEX did not differ from the wildtype (Figure 3A). Histochemical H_2_O_2_ visualization using 3,3′-diaminobenzidine (DAB) confirmed the above results (Figure 3B). Moreover, the differences in H_2_O_2_ content were accompanied by deregulated activity of superoxide dismutase (SOD). In mock-treated plants, only the *eds1*/Bax line demonstrated a significantly higher activity of SOD in relation to the wild type. Within DEX-treated plants, the highest SOD activity was found in *eds1* and *eds1*/Bax, while SOD activity in Bax + DEX plants did not differ from wildtype plants (Figure 3C). In addition, significant differences in the expression level of ROS marker genes (Table S2) [48,49] have been found (Figure 4A–C). *TRYPSIN INHIBITOR PROTEIN 1* (*TL1*, AT2G43510) was proposed as a marker gene for many ROS forms and *DMR6-LIKE OXYGENASE* 1 (*DLO1*, AT4G10500) is marker gene for H_2_O_2_, while beta-glucosidase BLG23 (AT3G09260) and *BIFUNCTIONAL INHIBITOR/LIPID-TRANSFER PROTEIN* (*BIF*, AT4G22490) are markers genes for superoxide (O_2_^-^) [48]. Moreover, *BON1-associated protein 1* (*BAP1*, AT3G61190) [50] and *AAA-ATPase 1* (*AAA-ATPase1*, AT3G28580) [51] were used as specific singlet oxygen (^1^O_2_) marker genes. Among the plants treated with the mock solution, only the Bax line demonstrated significantly higher TL1 expression level than the wildtype. In the group of plants treated with the DEX solution, *eds1* mutant plants showed significantly lower TL1 expression level, while Bax plants exhibited the highest expression level of TL1. Importantly, the level of TL1 expression in *eds1*/Bax did not differ from the wildtype (Figure 4A). The expression level of DLO1 in wildtype, *eds1*, and *eds1*/Bax plants was very low both under mock and DEX treatment. Meanwhile, DLO1 was six times more expressed in the Bax line treated with the mock solution and even higher (more than ten times) in the Bax + DEX line when compared to the wildtype (Figure 4B). Within mock-treated plants, the level of BLG23 expression was significantly induced in the *eds1* mutant and Bax line in relation to Col-0. In Bax plants treated with the DEX solution, BLG23 expression level was two times higher than in the wildtype (Figure 4C). However, the mutation in *eds1* reverted the BLG23-induced expression in Bax + DEX since there was no significant difference in BLG23 expression between the *eds1*/Bax + DEX line and Col-0 + DEX. We did not find any changes in BIF expression level among the wildtype, *eds1*, and *eds1*/Bax lines for non-treated or DEX-treated samples. However, a higher expression level of *BIF* was found in Bax plants both treated with mock and DEX (Figure 4D). Col-0, *eds1*, and *eds1*/Bax regardless of the treatment did not exhibit changed expression in specific markers genes for ^1^O_2_, while Bax plants exhibited very high expression of *BAP1* and *AAA**-**ATPase1* in both mock- and DEX-treated samples (Figure S3A,B). Our results clearly prove that EDS1 is necessary and is a crucial ROS homeostasis regulator during Bax-induced plant cell death.

### 3.3. EDS1 is Essential for the Induction of Hypersensitive Response and Senescence During Bax-Induced Plant Cell Death

**Figure 5:**
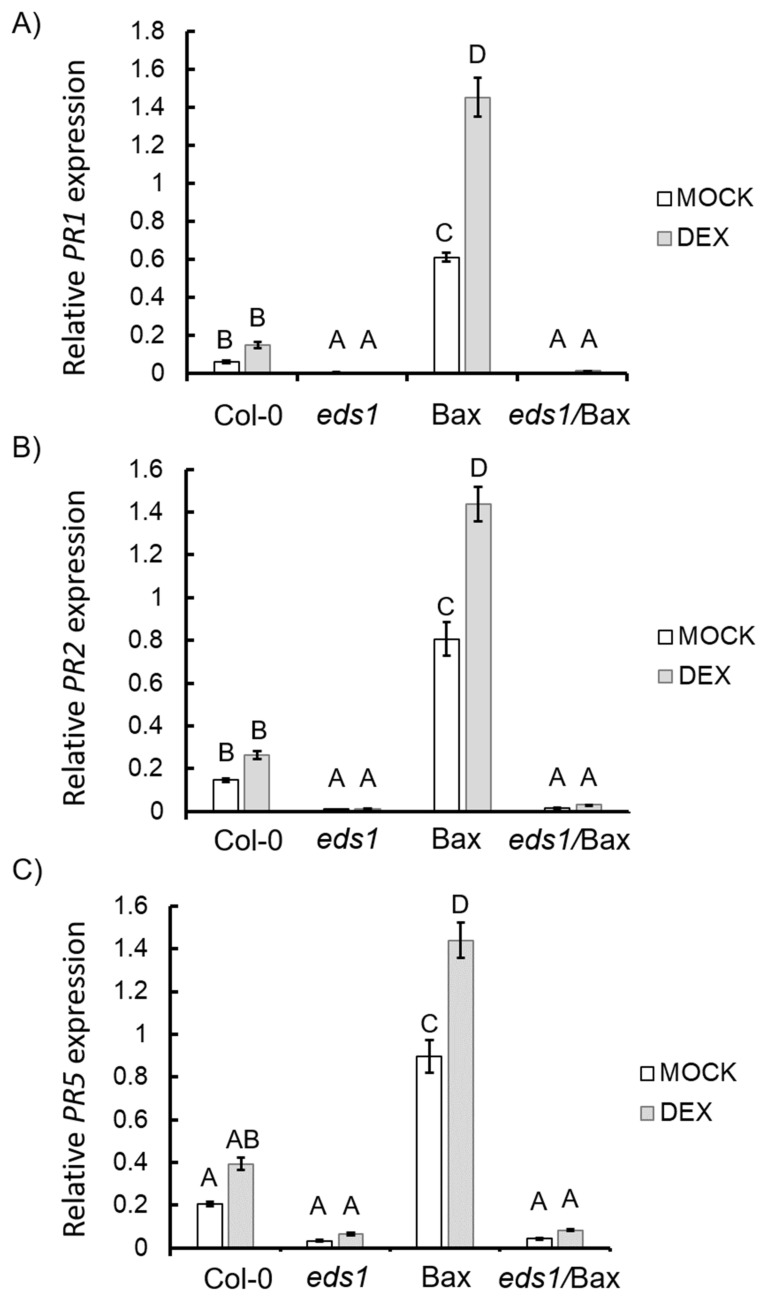
Relative expression levels of hypersensitive response (HR) marker genes in the wildtype, *eds1* mutant, and Bax and *eds1*/Bax lines: (**A**) *PR1*, (**B**) *PR2*, *and* (**C**) *PR5*. Within a subgraph, values sharing common labels (letters) are not significantly different from each other (*p* > 0.001) (*n* = 6).

EDS1 has been shown to positively regulate the expression level of HR marker genes, and the *eds1* mutant is unable to induce the expression of *PATHOGENESIS-RELATED (PR)* genes [52,53]. On the other hand, the overexpression of *EDS1* leads to higher PR genes expression [54]. Therefore, in the next step, we wanted to know if EDS1-dependent PR gene expression is involved in Bax-induced plant cell death. *PR1* (AT2G14610), *PR2* (AT3G57260), *PR5* (AT1G75040), *HIN1-LIKE 8* (*NHL8*, AT1G32340), and *SENESCENCE-ASSOCIATED* GENE 12 (*SAG12*, AT5G45890) [52] were used as hypersensitive response marker genes [6,8,52,53,54]. Among plants treated with the mock solution, the highest level of *PR* gene expression was exhibited by the Bax line (Figure 5A–C). The expression of *PR*-genes in the *eds1* mutant was marginal. We also found that the *eds1*/Bax line demonstrated a similarly low level of all PR gene expressions to the *eds1* mutant. Among plants treated with the DEX solution, we did not observe any significant changes in the expression level of *PR*-genes in wildtype, *eds1*, or *eds1*/Bax plants. On the contrary, the expression level of *PR1*, *PR2*, and *PR5* was significantly upregulated in Bax + DEX plants (Figure 5A–C). A similar level of *NHL8* expression was found in the wildtype, *eds1*, and *eds1*/Bax plants regardless of treatment. However, in the DEX-treated Bax line, the level of *NHL8* expression was upregulated in comparison to the wildtype (Figure S4A). The expression level of a senescence marker gene, *SAG12* [55], was significantly elevated in the Bax line, irrespective of mock or DEX treatment (Figure S4B). On the contrary, the *SAG12* expression level in both the mock- and DEX-treated wildtypes, in *eds1*, and in *eds1*/Bax plants was marginal (Figure S4B). These results clearly show that, during Bax-induced plant cell death, the HR is activated and that EDS1 is necessary for this process.

### 3.4. Arabidopsis thaliana Type I Metacaspases Seem to Be Involved in Bax-Induced Plant Cell Death

In mammalian cells, caspase activity is an essential step in Bax-dependent cell death [5]. True caspases have not yet been described in plants [56]; however, in plants, there are caspase-like proteins: metacaspases [57]. Therefore, we decided to check the expression level of genes encoding the two best-described metacaspases, METACASPASE *1* and *2* (*MC1* and *MC2*). The expression level of *MC1* was relatively high in all mock-treated genotypes. *MC1* was slightly upregulated in the Bax line and downregulated in the *eds1* mutant and *eds1*/Bax line; however, these changes were not statistically significant when compared to the wildtype (Figure S5A). Among plants treated with the DEX solution, we found higher *MC1* expression in Bax and *eds1*/Bax in comparison to the *eds1* mutant. The expression level of *MC2* was not significantly different in mock-treated *eds1* and *eds1*/Bax plants but significantly higher in the Bax line in comparison to the wildtype. In response to the DEX treatment, we found significant upregulation of the *MC2* expression level in the Bax line in relation to Col-0 + DEX. The *eds1* mutant demonstrated significant downregulation of *MC2* expression when compared to the wildtype, and the same tendency was shown in the *eds1*/Bax line.

### 3.5. Improved Antioxidant System and Impaired HR Result in the Mitigation of Mammalian Bax-Induced Cellular Organelle Destruction

**Figure 6:**
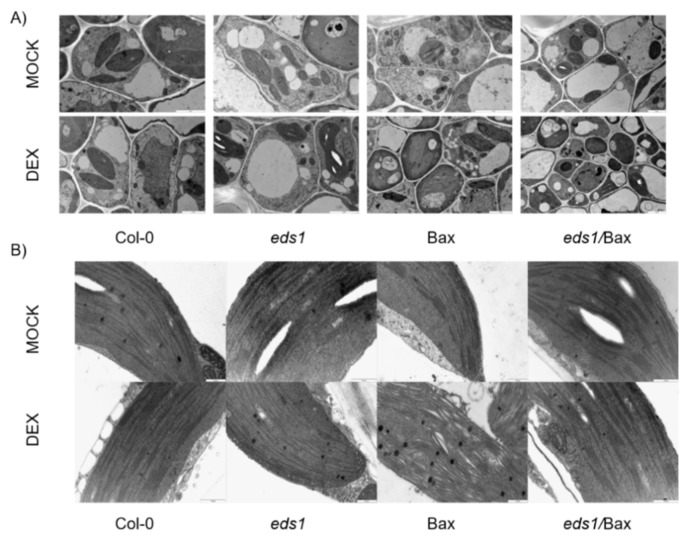
Transmission electron microscopy (TEM) images presenting cell architecture (**A**) and chloroplasts (**B**) in the wildtype, *eds1* mutant, and Bax and *eds1*/Bax lines (+mock/+DEX).

Among the plants treated with the mock solution, the anatomy of the leaves and the ultrastructure of leaf mesophyll cells were similar. In all tested cells, we found a central vacuole typical for mesophyll cells. Chloroplasts as well as mitochondria had typical shapes. The structure of thylakoids was not disturbed (Figure 6A). Wildtype and *eds1* mutant cells treated with the DEX solution looked the same as mock-treated plants, and we did not demonstrate any disturbances (Figure 6A). However, cells of the Bax line treated with DEX were clearly damaged. We observed a clear disintegration of the membranes and we found more plastoglobules that were bigger in comparison to the wild type. Moreover, the thylakoids were clearly distended (Figure 6A,B). In *eds1*/Bax cells, the damage was not so clear, and those cells looked more similar to the wildtype cells than the cells of the Bax line (Figure 6A). The main aberration in *eds1*/Bax cells was some damage within thylakoids (Figure 6B). Interestingly, we did not observe mitochondrial damage in any of the tested genotypes or treatments. These results indicate that, during Bax-induced cell death, EDS1 is involved in the process of cellular membranes disintegration, probably by its role in ROS and hormonal homeostasis regulation.

### 3.6. EDS1 Does Not Significantly Affects the Efficiency of PSII During Bax-Induced Plant Cell Death

No difference in Fv/Fm or Fv’/Fm’ was found among genotypes tested in this study, regardless of treatment (Figure S6A,B). The only difference found concerned ϕPSII, which was lower in the Bax line in comparison to other genotypes (Figure S6C). Interestingly, NPQ was significantly increased in the *eds1*/Bax line after DEX treatment, which was not observed in the Bax line (Figure S6D).

## 4. Discussion

It was suggested that direct comparison of Bax-induced plant cell death and HR-mediated cell death at the cellular level would provide valuable information concerning plant programmed cell death (PCD) and its similarity to mammalian apoptosis [17]. In this work, we confirmed previous reports indicating that mammalian Bax is able to induce cell death in plants [8,16,18]. So far, it was postulated that Bax-induced cell death in plants is similar to HR [8], occurs via ROS-dependent and -independent pathways [17], and is initiated by organelles destruction [7,16]. However, the molecular pathways and individual proteins involved in the Bax-dependent cell death pathway in plants were weakly described. Therefore, the aim of the current work was to test if EDS1, a plant-specific regulator of PCD, may be involved in Bax-induced plant cell death pathways. By crossing the *eds1* mutant with the Bax-expressing line (under a DEX-inducible promoter) [7] we obtained the *eds1*/Bax line, which was used to examine the role of EDS1 in Bax-induced ROS accumulation, membrane destruction, HR, and finally plant cell death.

In the course of our research, we found strong cell death symptoms in plants, where Bax was induced with DEX treatment. Bax + DEX plants were significantly smaller than wildtype plants, showed yellowing of the leaves, and exhibited elevated cell death level. However, we found no differences in rosette size of the *eds1*/Bax + DEX line in comparison to the wild type, and these plants did not exhibit characteristic, Bax-specific yellowing. In *eds1*/Bax + DEX plants, we found a slightly higher level of cell death in comparison to the wild type but still a significantly lower one than in Bax + DEX plants. It was previously found that mammalian Bax-induced cell death in plants can be downregulated by *BI-1* overexpression [7,58]. However, in *BI-1*-OE/Bax plants, the cell death phenotype was not completely reversed and *PR1* protein and ROS were still over-accumulated [16,18,58]. Those plants still exhibited reduced size, increased chlorophyll degradation, and higher cell death level [16,58]. Such a strong phenotype reversal in *eds1*/Bax plants in terms of cell death is caused by significant changes in HR, by ROS homeostasis regulation, and by changes within membranes integration, which were all previously proposed as Bax-dependent cell death features in plants [16,17,58].

One pathway of Bax-induced HR occurs through ROS signalling [17]. It is known that ROS can act as HR-triggering molecules [46,59] and that H_2_O_2_ induces *EDS1* expression [60]. In our study, we found that Bax + DEX plants exhibited a high H_2_O_2_ level in tissues and that the H_2_O_2_ level in *eds1*/Bax + DEX was significantly lower in comparison to Bax + DEX plants. Moreover, we found significant changes in the expression level of different ROS form markers. The expression of all *TL1*, *DLO1*, *BLGU23*, *BIF*, *BAP1*, and *AAA-ATPase1* was upregulated in Bax plants in comparison to the other genotypes. Higher expression of ^1^O_2_ marker genes could be causes by certain disturbances in photosystem II (Figure S6). Studying the ROS marker genes, we showed that, during Bax-induced plant cell death, many ROS forms can be overproduced and that EDS1 is involved in their metabolism, scavenging, or signalling [61]. Moreover, in our study, Bax + DEX plants demonstrated higher *EDS1* expression, compared to the other genotypes (Figure S1). In plant cells, the Bax protein is located in MOM, similar to animal cells [16,58]. Moreover, in mammalian cells, it was found that H_2_O_2_ is overproduced in mitochondria during apoptosis [62]. In our study, during Bax-induced plant cell death, we observed more significant damages within chloroplasts than in mitochondria [16,58]. Chloroplast damage can lead to further ROS production because of PSII disruption, which may be indicated by the inability to increase the NPQ, observed in the Bax line [63,64,65]. In the *eds1*/Bax + DEX line, there was no *EDS1* expression and lack of EDS1 inhibited HR induction also via the inhibition of ROS accumulation. EDS1 was postulated as an antioxidant system inhibitor [22], and it was found that the *Arabidopsis thaliana eds1* mutant accumulated less H_2_O_2_ in response to stress than the wildtype [20,47]. Moreover, lower H_2_O_2_ content and higher antioxidant system activity were found in *EDS1*-silenced transgenic poplar [66], which indicates that the role of EDS1 in ROS homeostasis is strongly preserved in higher plants.

HR is a plant response to a wide range of stresses, both biotic [67,68] and abiotic [69,70]. It is a multicomponent response manifested in higher *PR* genes expression, antimicrobial secondary metabolite accumulation, and lesion formation [46,68]. It was postulated that mammalian Bax-induced plant cell death is similar to HR, and it was found that the PR1 protein is over-accumulated when mammalian Bax is expressed in plants [8]. In present study, after Bax induction, we found significantly higher *PR1*, *PR2*, and *PR5* gene expressions. Moreover, we found higher *NHL8* and *SAG12* gene expression, which are proposed as early HR and senescence marker genes, respectively [52]. EDS1 and its interacting partner, PAD4, were described as important HR regulators [32,71]. It was shown that EDS1 is crucial in HR induction in response to *Botrytis cinerea* [72] and that harpin-elicited hypersensitive cell death depends on EDS1 [73]. In the wildtype tomato and Arabidopsis, it was found that the expression of *EDS1* is induced in response to biotic stress, which leads to higher *PR1* expression [32,72], while in the Arabidopsis *eds1* mutant, the *PR1* gene expression is significantly lower in comparison to the wildtype [71]. Moreover, it was demonstrated that the *eds1* mutation, introduced into the constitutive expressor of *PR genes 1* (*cpr1*) mutant, reverses the accumulation of *PR1*, *PR2*, and *PR5* mRNAs, characteristic for *cpr1* single mutant [53,73]. Likewise, it is known that HR is generally controlled by disease resistance (R) genes [66,68] and that EDS1 is required for the activity of the TIR-NBS-LRR class of R-genes [74]. It indicates that plants lacking the EDS1 protein are unable to activate the HR response [75,76]. In our study, we found a strong expression of *PR* genes in Bax + DEX and lack of or minimal *PR*-gene expression in *eds1*/Bax plants + DEX. Moreover, in *eds1*/Bax + DEX plants, there was no difference in *NHL8* expression level in comparison to the wildtype. These results prove that the expression of mammalian Bax in plant cells leads to HR induction and that a lack of active s inhibits this process.

Interestingly, Arabidopsis HR can be induced by metacaspases [77]. It is known that, in animal cells, Bax protein leads to the outflow of cytochrome C from the mitochondria to the cytoplasm, leading to caspase activation, which is a signal to initiate apoptosis [11,14,15]. It cannot be excluded that a similar mechanism occurs in plants. In our study, we found slightly higher *MC1* expression in both the Bax + DEX and *eds1*/Bax + DEX plants. Proteins encoded by *MC1* are a positive regulator HR, and it was shown that mutation in *MC1* nearly eliminates HR in Arabidopsis [77]. This result suggests that the Bax protein is active in both Bax + DEX and *eds1*/Bax + DEX plants but the lack of active EDS1 protein inhibits HR induction. It was also found that *MC1* pro-cell death activity is negatively regulated by LSD1 [77]. A direct interaction between LSD1 and EDS1 was previously demonstrated [35] so we can hypothesize that EDS1 is also involved in MC1-dependent HR induction. However, based on our results, we cannot say with certainty that Bax expression in plant cell leads to metacaspase activation in a similar way as caspase activation in animal cells. To confirm this, further studies are required. In addition, we found a significantly higher *MC2* expression level in Bax + DEX plants in comparison to other tested genotypes, including *eds1*/Bax + DEX. However, *MC2* is a negative regulator of cell death [75], and high expression of this gene could be linked to stress-related signalling pathways connected with Bax expression.

It was previously found that the expression of mammalian Bax in plant cells leads to organelle destruction [16,58]. In plants expressing mammalian Bax, the cytoplasmic shrinkage, chloroplast destruction, and changes in mitochondrial structures in mesophyll cells were apparent [58]. Interestingly, the disruption of chloroplasts was observed 1 to 2 days after Bax induction [16]. It indicates that chloroplast damage during Bax-induced cell death is caused by a secondary effect, most probably induced by ROS coming from mitochondria. It was found that the overexpression of *BI-1* mitigates organelle destruction when mammalian Bax is expressed in plants [58], but this mechanism has not been precisely explained. It was proposed that *BI-1* suppresses Bax-induced plant cell death acting downstream of oxidative burst or alternative processes lacking the involvement of ROS [17]. It is possible that BI-1 acts as a suppressor of the Bax-induced HR-like cell death in its early stage because it was found that *BI-1* is upregulated in response to many stresses and that the *atbi-1* mutant exhibited strong HR phenotype [19,78]. In our study, we found no differences between the mitochondria structure of Bax + DEX and *eds1*/Bax + DEX plants. However, the plasma membrane in Bax + DEX was clearly damaged, and strong changes in the thylakoids structure were found. These changes did not occur in *eds1*/Bax + DEX plants, and we propose that this is because of the improved antioxidant system in *eds1*/Bax plants. It could be another reason for strong reversal of the cell death phenotype and senescence symptoms in the *eds1*/Bax + DEX line. The senescence symptoms, such as membrane degradation, increased the number of plastoglobules and chloroplast degradation [79], which we found in Bax + DEX plants were not observed in *eds1*/Bax + DEX.

**Figure 7:**
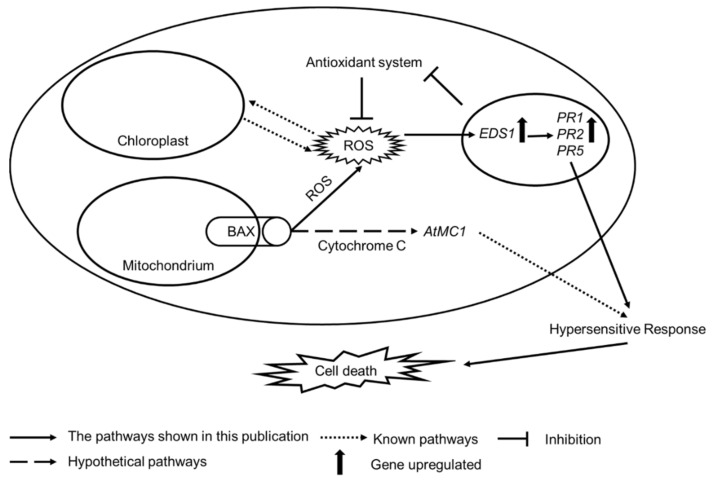
Proposed model of plant (EDS1) and animal (Bax) proapoptotic dependency in the cell death process in an artificial system used in this study: analogous to animal cells, the Bax protein in plant cells is located in the mitochondrial membrane [8,17]. It leads to the outflow of mitochondrial content (including ROS) to the cytoplasm. This so-called first ROS wave can act not only as a signal but also as a damaging factor to the chloroplast membranes, leading to the generation of a second ROS wave derived from chloroplasts. H_2_O_2_ induces *EDS1* expression in the nucleus [60]. EDS1 is required for PATHOGENESIS-RELATED (PR) protein accumulation. PR proteins trigger HR and, as a consequence, cell death [32]. Moreover, EDS1 acts as a negative regulator of the antioxidant system and thus contributes to increasing ROS content in plant tissues [22]. Furthermore, expression of mammalian Bax may lead to cytochrome C outflow to the cytoplasm and the activation of plant metacaspases. Some metacaspases, such as AtMC1, induce cell death.

Based on the previous reports and results described in the current work, we propose the following model of similarities in the molecular mechanism of cell death regulation in plants and animals and the involvement of plant protein EDS1 in the propagation of mammalian Bax-induced cell death (Figure 7). It was found that the Bax protein in plant cells, similar to animal cells, is located in the mitochondrial membrane [16,80]. Its main role is to allow the outflow of cytochrome C into the cytoplasm [81], but it has also been shown that Bax stimulates ROS production in mitochondria [81]. Cytoplasmic ROS causes damage to chloroplasts [82,83] and contributes to higher ROS production [84,85]. Moreover, it was found that ROS can induce the expression of *EDS1* in the nucleus ([72] and Figure S1). The EDS1 protein can support mammalian Bax-induced plant cell death in two ways. One scenario is that EDS1 acts as a negative regulator of the antioxidant system, which contributes to disturbance in ROS scavenging [22]. The second scenario is that EDS1 propagates mammalian Bax-induced plant cell by its role in HR, systemic acquired resistance (SAR), and systemic acquired acclimation (SAA) [8], which leads to cell death in Bax-expressing plants.

In this study, we demonstrate that the inability to initiate HR and improve ROS metabolism via mutation in *EDS1* nearly eliminates the cell death phenotype caused by the expression of mammalian Bax in plant cells. It indicates that EDS1 is functionally involved in Bax-induced cell death. However, it is still unknown how the HR is induced during Bax expression in plants, and more studies are required to explain this. Most importantly, we show that some of the molecular mechanism of cell death regulation are strongly preserved among living organisms and that they probably developed before the division of living organisms into plants and animals.

## Supplementary Materials

The following are available online at https://www.mdpi.com/2073-4409/9/11/2454/s1. Supplementary Figure 1. The relative expression level of (A) *EDS1* and (B) Bax in plants used in this study: within a subgraph, values sharing common labels (letters) are not significantly different from each other (*p* > 0.001) (*n* = 9). Supplementary Figure 2. Phenotype of the wildtype, *eds1* mutant, and Bax and *eds1*/Bax lines (+mock/+DEX). Supplementary Figure 3**.** Relative expression level of specific singlet oxygen (^1^O_2_) marker genes in the wildtype, *eds1* mutant, and Bax and *eds1*/Bax lines for (A) *BAP1* and (B) *AAA-ATPase1*: within a subgraph, values sharing common labels (letters) are not significantly different from each other (*p* > 0.001) (*n* = 9). Supplementary Figure 4. Relative expression level of hypersensitive response (HR) marker genes in the wildtype, *eds1* mutant, and Bax and *eds1*/Bax lines for (A) *NHL8* and (B) *SAG101*: within a subgraph, values sharing common labels (letters) are not significantly different from each other (*p* > 0.001) (*n* = 9). Supplementary Figure 5. The relative expression level of (A) *MC1* and (B) *MC2*: within a subgraph, values sharing common labels (letters) are not significantly different from each other (*p* > 0.001) (*n* = 8–10). Supplementary Figure 6. Chlorophyll fluorescence parameters: (A) photochemistry in the dark-adapted state (Fv/Fm), (B) maximum quantum efficiency of photosystem II (PSII) photochemistry (Fv’/Fm’), (C) effective quantum yield of PSII photochemistry (ϕPSII), and (D) non-photochemical quenching (NPQ). Within a subgraph, values sharing common labels (letters) are not significantly different from each other (*p* > 0.05) (*n* = 18–22). Supplementary Table 1. List of primers used in this study. Supplementary Table 2. ROS marker genes used in this study.
