# Supplementary material for: EDS1-Dependent Cell Death and the Antioxidant System in Arabidopsis Leaves is Deregulated by the Mammalian Bax
Source: Cells. 2020 Nov 10;9(11):2454. doi: 10.3390/cells9112454 (PMC7698216; doi:10.3390/cells9112454)
Supplement: Supplementary file 1 [file cells-09-02454-s001.pdf]

Supplementary Data

EDS1-Dependent Cell Death and the Antioxidant System in *Arabidopsis* Leaves is Deregulated by the Mammalian Bax

Maciej Jerzy Bernacki [1,], Weronika Czarnocka [2,3], Magdalena Zaborowska [2], Elżbieta Różańska [3], Mateusz Labudda [4], Anna Rusaczonek [2,3], Damian Witoń [2] and Stanisław Karpiński [2,*]

[1]Institute Of Technology And Life Sciences, Falenty, Al. Hrabska 3, 05-090 Raszyn, Poland
[2]Department of Plant Genetics, Breeding and Biotechnology, Institute of Biology, Warsaw University of Life Sciences, Nowoursynowska Street 159, 02-776 Warszawa, Poland
[3]Department of Botany, Institute of Biology, Warsaw University of Life Sciences, Nowoursynowska Street 159, 02-776 Warszawa, Poland
[4] Department of Biochemistry and Microbiology, Institute of Biology, Warsaw University of Life Sciences, Nowoursynowska Street 159, 02-776 Warszawa, Poland

* Correspondence: stanislaw_karpinski@sggw.edu.pl

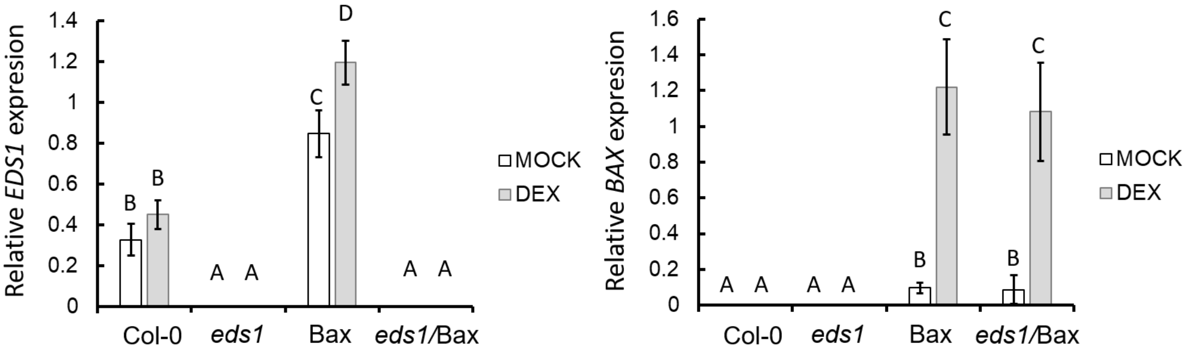

Supplementary Figure 1. The relative expression level of (**A**) *EDS1* and (**B**) *Bax* in plants used in this study. Within a subgraph, values sharing common labels (letters) are not significantly different from each other ($p > 0.001$) ($n = 9$).

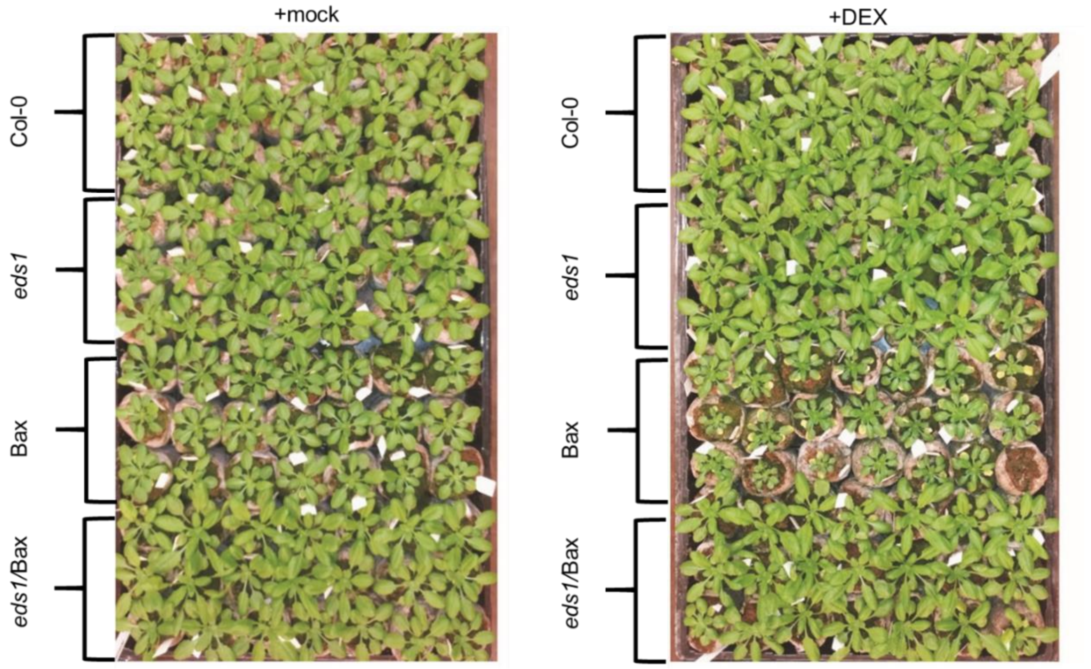

Supplementary Figure 2. Phenotype of the wild type, *eds1* mutant, Bax and *eds1*/Bax lines (+MOCK/+DEX).

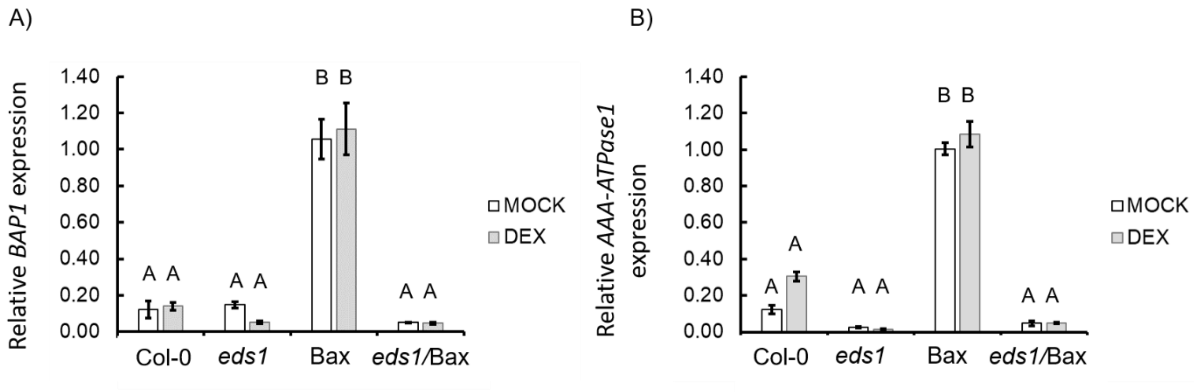

Supplementary Figure 3. Relative expression level of specific singlet oxygen ($^1O_2$) marker genes in the wild type, *eds1* mutant, Bax and *eds1*/Bax lines (**A**) *BAP1* and (**B**) *AAA-ATPase1*. Within a subgraph, values sharing common labels (letters) are not significantly different from each other ($p > 0.001$) ($n = 9$).

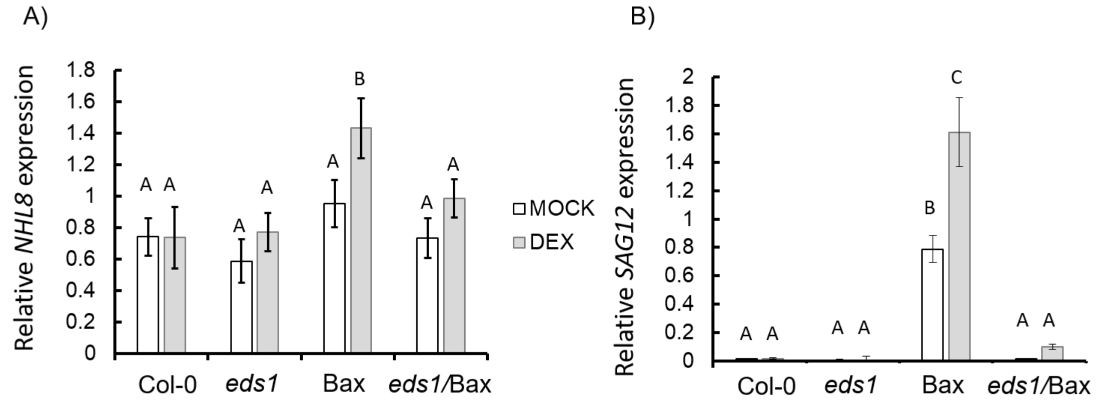

Supplementary Figure 4. Relative expression level of hypersensitive response (HR) marker genes in the wild type, *eds1* mutant, Bax and *eds1*/Bax lines (**A**) *NHL8* and (**B**) *SAG101*. Within a subgraph, values sharing common labels (letters) are not significantly different from each other ($p > 0.001$) ($n = 9$).

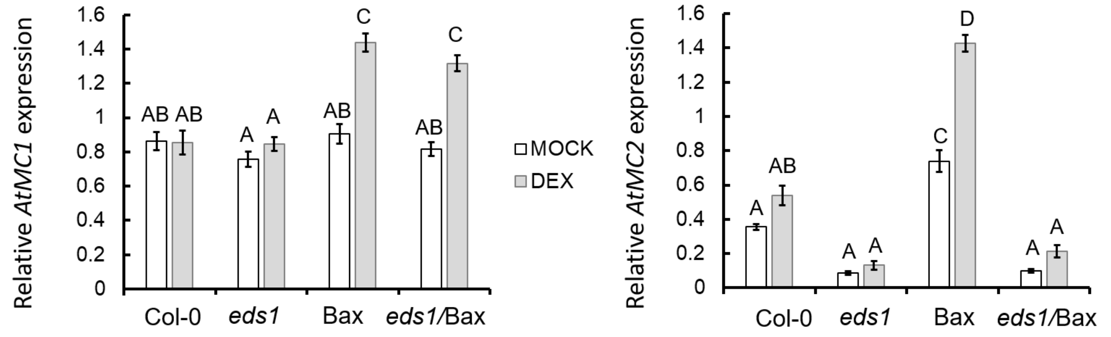

Supplementary Figure 5. The relative expression level of (**A**) *MC1* and (**B**) *MC2*. Within a subgraph, values sharing common labels (letters) are not significantly different from each other ($p > 0.001$) (n = 8-10).

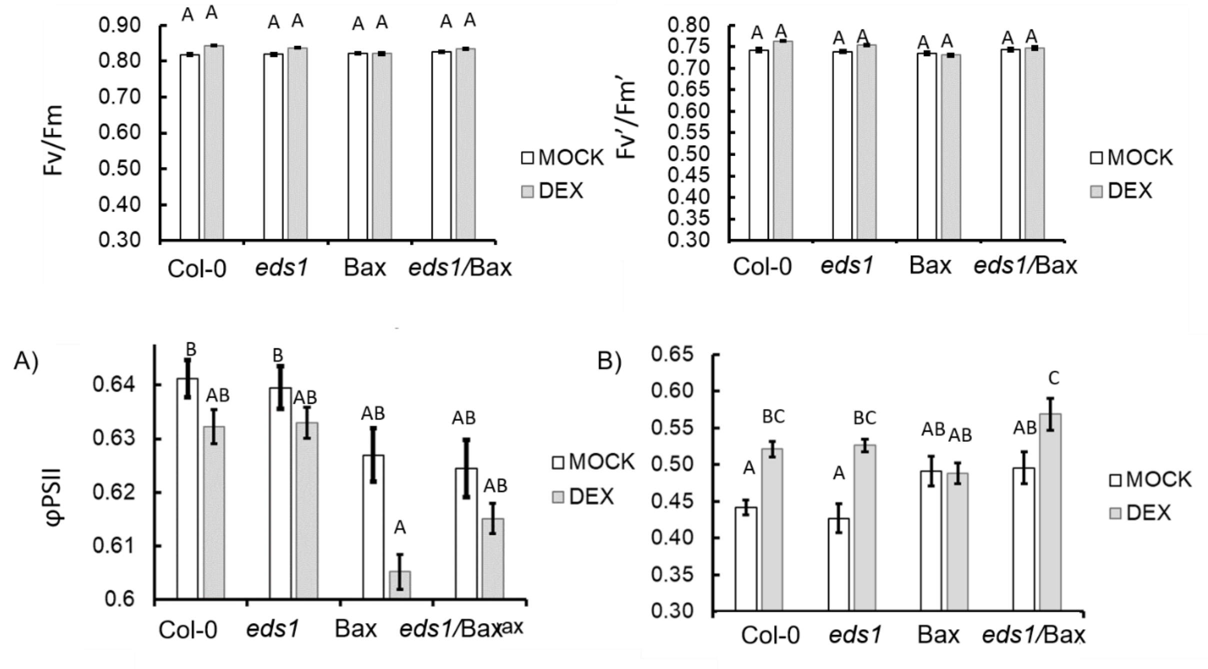

Supplementary Figure 6. Chlorophyll fluorescence parameters. (**A**) photochemistry in the dark adapted state (Fv/Fm), (**B**) maximum quantum efficiency of PSII photochemistry (Fv'/Fm'), (**C**) effective quantum yield of PSII

photochemistry (ϕPSII), and (**D**) non-photochemical quenching (NPQ). Within a subgraph, values sharing common labels (letters) are not significantly different from each other ($p > 0.05$) (n = 18-22).

Supplementary Table 1. List of primers used in this study.

	Gene abbreviation	AGI code	Forward primer	Reverse primer
1	*5-FCL*	AT5G13050	GCAAACTCAATGAACATTTTGG	GATCGGTTCATCTGCTTGC
2	*PP2A*	AT3G25800	TAACGTGGCCAAAATGATGC	GTTCTCCACAACCGCTTGGT
3	*BAX*		TGCAGAGGATGATTGCTGAC	GATCAGCTCGGGCACTTTAG
4	*EDS1*	AT3G48090	CTCAATGACCTTGGAGTGAGC	TCTTCCTCTAATGCAGCTTGAA
5	*TI1*	AT2G43510	TCGTTTCCATCTTCGTTGTCT	GCTTCTATCTCCGGCACATC
6	*BGLU23*	At3g09260	AACACAGACGCCTTTAGAATGTC	GAATTGCACACCAGCTTGAC
7	*BIF*	At4g22490	CAACCACCATTTCCCTCATC	GCGAGCTTAAGAGGTTTGCTT
8	*DLO1*	At4g10500	CCCAACCGTTATCACTGTCC	AGCGACCCATTTATCATCCTT
9	*BAP1*	AT3G61190	TAAACCGGAGACCCATCAAG	TTCGACATTTCTCGTCGATTT
10	*AAA-ATPase1*	AT3G28580	CTGACGATTTTGAGGGCATT	GCTTCAGGGTAAAACGAGAAAG
11	*PR1*	AT2G14610	TTCTTCCCTCGAAAGCTCAA	GCCTGGTTGTGAACCCTTAG
12	*PR2*	AT3G57260	TCTCCCTTGCTCGTGAATCT	CGTGTCTCCCATGTAGCTGA
13	*PR5*	AT1G75040	CGTACAGGCTGCAACTTTGA	CTTAGACCGCCACAGTCTCC
14	*NHL8*	AT1G32340	TCCAGTCAACGATCATCCAA	AAATCCGTCGAAGTCGTGTC
15	*AtMC1*	AT1G02170	CAACCATTGTACGCCCTCTT	ACGGTACCACTATGGCAAGC
16	*AtMC2*	AT4G25110	CAACATAACAATGGCGATGC	GACCGGAGAAGTGAAAGACG
17	*SAG12*	AT5G45890	CTCGTCCACTCGACAATGAA	CCACTCGATGTGCCTCTTTT

Supplementary Table 2. ROS marker genes used in this study.

	Gene abbreviation	AGI code	Description
1	*TI1*	AT2G43510	Up regulated in response to mean ROS forms
2	*BGLU23*	AT3G09260	Transcripts specifically responsive to superoxide
3	*BIF*	AT4G22490	Transcripts specifically responsive to superoxide
4	*DLO1*	AT4G10500	Transcripts specifically responsive to hydrogen peroxide
5	*BAP1*	AT3G61190	Transcripts specifically responsive to singlet oxygen
6	*AAA-ATPase1*	AT3G28580	Transcripts specifically responsive to singlet oxygen
